# COVID-19 Pandemic and Vaccines Update on Challenges and Resolutions

**DOI:** 10.3389/fcimb.2021.690621

**Published:** 2021-09-10

**Authors:** Wajihul Hasan Khan, Zohra Hashmi, Aditya Goel, Razi Ahmad, Kanisha Gupta, Nida Khan, Iqbal Alam, Faheem Ahmed, Mairaj Ahmed Ansari

**Affiliations:** ^1^Department of Biotechnology, Host Pathogen Interaction and Molecular Immunology Laboratory, Jamia Hamdard, New Delhi, India; ^2^Department of Chemical Engineering, Indian Institute of Technology Delhi, New Delhi, India; ^3^Department of Chemistry, Indian Institute of Technology Delhi, New Delhi, India; ^4^Department of Physiology, Hamdard Institute of Medical Sciences and Research, Jamia Hamdard, New Delhi, India; ^5^Department of Community Medicine, Hamdard Institute of Medical Sciences and Research, New Delhi, India

**Keywords:** COVID-19, SARS CoV-2 variant, spike protein, mutations, immune response, vaccine, multivariant vaccines, booster dose

## Abstract

The coronavirus disease (COVID-19) is caused by a positive-stranded RNA virus called severe acute respiratory syndrome coronavirus-2 (SARS-CoV-2), belonging to the *Coronaviridae* family. This virus originated in Wuhan City, China, and became the cause of a multiwave pandemic that has killed 3.46 million people worldwide as of May 22, 2021. The havoc intensified with the emergence of SARS-CoV-2 variants (B.1.1.7; Alpha, B.1.351; Beta, P.1; Gamma, B.1.617; Delta, B.1.617.2; Delta-plus, B.1.525; Eta, and B.1.429; Epsilon etc.) due to mutations generated during replication. More variants may emerge to cause additional pandemic waves. The most promising approach for combating viruses and their emerging variants lies in prophylactic vaccines. Several vaccine candidates are being developed using various platforms, including nucleic acids, live attenuated virus, inactivated virus, viral vectors, and protein-based subunit vaccines. In this unprecedented time, 12 vaccines against SARS-CoV-2 have been phased in following WHO approval, 184 are in the preclinical stage, and 100 are in the clinical development process. Many of them are directed to elicit neutralizing antibodies against the viral spike protein (S) to inhibit viral entry through the ACE-2 receptor of host cells. Inactivated vaccines, to the contrary, provide a wide range of viral antigens for immune activation. Being an intracellular pathogen, the cytotoxic CD8^+^ T Cell (CTL) response remains crucial for all viruses, including SARS-CoV-2, and needs to be explored in detail. In this review, we try to describe and compare approved vaccines against SARS-CoV-2 that are currently being distributed either after phase III clinical trials or for emergency use. We discuss immune responses induced by various candidate vaccine formulations; their benefits, potential limitations, and effectiveness against variants; future challenges, such as antibody-dependent enhancement (ADE); and vaccine safety issues and their possible resolutions. Most of the current vaccines developed against SARS-CoV-2 are showing either promising or compromised efficacy against new variants. Multiple antigen-based vaccines (multivariant vaccines) should be developed on different platforms to tackle future variants. Alternatively, recombinant BCG, containing SARS-CoV-2 multiple antigens, as a live attenuated vaccine should be explored for long-term protection. Irrespective of their efficacy, all vaccines are efficient in providing protection from disease severity. We must insist on vaccine compliance for all age groups and work on vaccine hesitancy globally to achieve herd immunity and, eventually, to curb this pandemic.

## 1. Introduction

Coronaviruses are named after the crown-like spikes present on their outer surfaces. Out of seven known human coronaviruses (HCoVs), four viruses, HCoV-229E (alphacoronavirus genus), HCoV-NL63 (alphacoronavirus genus), HCoV-OC43 (betacoronavirus genus), and HCoV-HKU1 (betacoronavirus genus), cause mild upper respiratory tract disease in adults. However, SARS-CoV and Middle East respiratory syndrome coronavirus (MERS-CoV) caused pandemic in 2002–2003 and 2012, respectively (Zeng et al., 2018; Chakraborty et al., 2020). The SARS-CoV-2, a seventh member of the HCoV family, is an etiological agent of the COVID-19 pandemic as announced by WHO on March 11, 2020 (Cucinotta and Vanelli, 2020). Surprisingly, all pandemic-causing HCoVs, including SARS-CoV-2, belong to the betacoronavirus genus and are zoonotic in nature (Mackenzie and Smith, 2020; Piret and Boivin, 2021).

**Figure 1:**
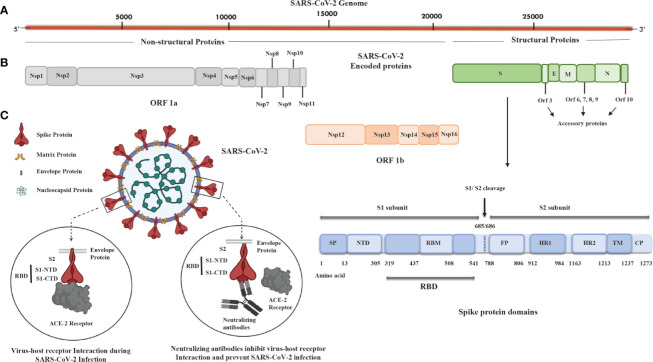
SARS-CoV-2 genome, encoded proteins, and basic mechanism of virus fusion and entry. **(A)** Illustration of the SARS-CoV-2 genome that is around 30 kb in size and has a 5’ cap and 3’ poly A tail. **(B)** SARS-CoV-2 proteins: nonstructural proteins (Nsp), ORF1a, and ORF1b (Nsp1-Nsp16) and structural proteins such as spike (S), envelope (E), membrane (M), and nucleocapsid (N) with spike protein having 1273 aa (~180 kDa) containing signal peptide (SP), N-terminal domain (NTD), receptor binding motif (RBM) in receptor binding domain (RBD), fusion peptide (FP), heptad repeat (HR)-1, HR-2, transmembrane domain (TM), and cytoplasmic tail (CP) domains. There are accessory proteins Orf3; Orf6,7,8,9; and Orf10 located in between the S, E, M, and N proteins. **(C)** The spike protein subunit 1 (S1; aa 14-685) of SARS-CoV-2 consists of the RBD domain divided into two parts: S1 N-terminal domain (S1-NTD) and S1 C-terminal domain (S1-CTD) and spike protein subunit 2 (S2; aa 686-1273) containing TM and CP domains. S1-CTD interacts with the angiotensin-converting enzyme-2 (ACE-2) receptor of host cells and facilitates fusion and entry of virus. The neutralizing antibody against S1-CTD blocks the entry of the SARS-CoV-2 into host cells. ORF1a; Open reading frame 1a, ORF1b; Open reading frame 1b, aa; Amino acid.

In light of the evolutionary linkages of SARS-CoV-2, its genomic structures coincide with other HCoVs in the range of 65.04% to 82.45% with SARS-CoV showing the highest similarity (Gorbalenya et al., 2020; Nakagawa and Miyazawa, 2020). SARS-CoV-2 is extremely infectious and transmissible due to its high propensity for attaching to angiotensin-converting enzyme-2 (ACE-2) receptors of the host cells, resulting in its quick spread from the epicenter in Wuhan, China, to more than 200 countries worldwide. It can be transmitted directly by contacting an infected person’s respiratory droplets or indirectly by coming into contact with anything used or touched by an infected person (Kanamori et al., 2020). COVID-19 symptoms include anything from a simple respiratory infection to severe pneumonia, acute respiratory distress syndrome (ARDS), hypoxia, multiorgan failure, and death. Additionally, lymphopenia is also reported in a meta-analysis study of COVID-19 patients and is linked with a higher mortality, ARDS, severe clinical symptoms, and ICU admission. It is recorded that patients who died from COVID-19 had significantly lower lymphocyte counts than survivors (Ruan et al., 2020). Because of the similitudes with SARS-CoV, the international committee on taxonomy of viruses named the novel virus SARS-CoV-2 (Gorbalenya et al., 2020; Kumar et al., 2021). SARS-CoV-2 is a spherical or pleomorphic wrapped particle containing single-stranded positive-sense RNA associated with a nucleoprotein inside a capsid (Mousavizadeh and Ghasemi, 2021). The envelope provides a platform for club-framed glycoprotein projections. Some coronaviruses additionally contain a hemagglutinin-esterase protein (HE). The RNA genome of size around 30 kb encodes 16 nonstructural proteins (Nsp1 to Nsp16) and four structural proteins named spike (S), envelope (E), membrane (M), and nucleocapsid (N) (**Figures 1A, B**). Open reading frame (ORF)1ab (265-21555 bp) encodes polyproteins PP1ab (codes for Nsp1 to Nsp16) or shorter proteins (PP1a; codes for Nsp1 to Nsp11), depending on a 1 ribosomal frameshift event. The polyproteins are cleaved either by papain-like proteinase protein (PLpro) or main protease (Mpro) to yield 16 nonstructural proteins (Gioia et al., 2020). Out of the 16 nonstructural proteins, PLpro (Nsp3), 3C-like proteinase (Nsp5, Mpro), RNA-dependent RNA polymerase (Nsp12, RdRp), helicase (Nsp13), endoRNAse (Nsp15), 2’-O-Ribose-Methyltransferase (Nsp16), and other nonstructural proteins show similarity with other coronaviruses. However, the remaining 3’ genome codes for S (~180 kDa glycoprotein), E (9-12 kDa protein), M (23-35 kDa protein), and RNA binding basic N protein (~45.6 kDa). Apart from structural proteins, the 3’ end also encodes nine accessory proteins, Orf3a, Orf3b, Orf6, Orf7a, Orf7b, Orf8, Orf9b, Orf9c, and Orf10, and these proteins are involved in viral replication, assembly, and egress; they also have the potential to serve as antigens (Michel et al., 2020). The S protein trimerizes to provide the viral crown structure, making it a highly exposed protein of the virus. Thus, the trimeric S antigen is a major protective antigen that elicits highly potent neutralizing antibodies (Yang et al., 2020). The spike protein is 1273 amino acid glycoprotein mainly composed of two major subunits: S1 (aa 14-685) and S2 (aa 686-1273). At the N terminal of the S1 subunit, there is a signal peptide (SP; aa 1-13); the S1 subunit contains the N terminal domain (NTD, aa 14-305) and receptor binding domain (RBD; aa 319-541), and the RBD contains RBM (aa 437-508) and a C terminal domain (CTD) (**Figure 1**). The RBD of the S1 subunit binds to the ACE-2 receptor of the host cell. However, the S2 subunit responsible for fusion and entry have fusion peptides (FP; aa 788-806), heptapeptide repeat sequence-1 (HR-1; aa 912-984), HR-2 (aa 1163-1213), transmembrane domains (TM; aa 1213-1237), and cytoplasmic tail (CP; aa 1237-1273) domains (**Figure 1B**). Based on the important role of the spike protein in viral transmission, most of the vaccines, including adenovirus-, mRNA-, and DNA-based candidates, are generating neutralizing antibodies against the spike protein to block viral entry (**Figure 1C**) (De Haan et al., 1998). On the other hand, the inactivated virus provides all the antigens; hence, the immune responses developed in vaccinated individuals are not only against the spike but against numerous viral antigens as well. Other than the S protein, the N, M, nonstructural proteins (nsps) and accessory proteins may also have antigenic potential to serve as a candidate vaccine (Blanco-Melo et al., 2020). In fact, promising antigenic determinants (epitopes) in M, N, and S proteins, 4, 8, and 13 epitopes, respectively, have been identified using an extensive immuno-informatics analysis of SARS-CoV-2 proteins. Among these, M protein (165–181 and 306–322), N protein (314–330), and S protein (817–833, 891–907, 897–913, and 1182–1209) were found to be nonallergenic and nontoxic and have a low chance of producing autoimmune reactions in 87% of the world’s population (Mukherjee et al., 2020). These antigens could be used for developing effective candidate vaccines against SARS-CoV-2.

**Figure 2:**
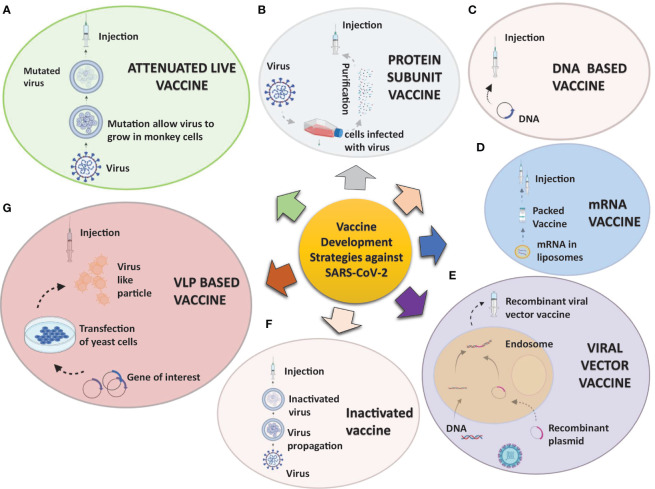
Strategies being utilized to develop vaccine candidates against SARS-CoV-2: A concise overview of various aspects of the SARS-CoV-2 vaccine development process varying from traditional to novel platforms. **(A)** Attenuated live pathogen vaccine: A debilitated (infection incompetent) form of the live pathogen obtained by lengthy cell culture passaging in nonhuman cell lines or animals are administered. **(B)** Protein subunit vaccines are prepared from either antigen purification of pathogens replicated in cell cultures or recombinant expressed antigens. **(C, D)** Nucleic acid vaccines: mRNA **(C)** or DNA **(D)** codifying for an immunogenic protein of the pathogen express and present the antigen in antigen-presenting cells. The mRNA is mixed with nanoparticles or other stabilizing agents, and DNA is inserted in a vector. **(E)** Viral vector vaccines: Recombinant viral vectors are produced by genetic manipulation of measles or the adenoviral platform to express the antigen of interest. **(F)** Inactivated pathogen vaccines contain the whole pathogen that has been subjected to heat or chemical treatment for inactivation. **(G)** Virus-like particles vaccines: These are virus-like particles (20–200 nm) assembled and released by many baculoviruses or the mammalian expression system, e.g., recombinant yeast cells, vaccinia virus expression system, or even tobacco plants transfected with tobacco mosaic virus platform.

**Figure 3:**
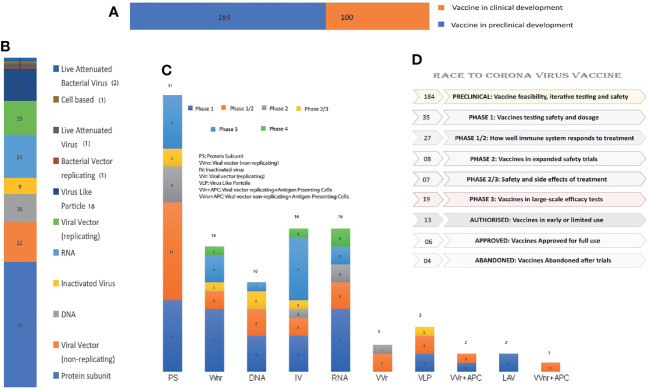
Summary and number distribution of candidate vaccines in preclinical and clinical trials: **(A)** Clinical stages of candidate vaccines: Number of vaccines under clinical (depicted by orange color; 100) and preclinical development (depicted by blue color; 184). **(B)** Candidate vaccines of different platforms under clinical trial from bottom to top: Protein Subunit, VVnr, DNA, Inactivated Virus, RNA, VVr, VLP, BVr, LAV, Cell based and LABv with their respective candidate vaccine numbers. **(C)** Candidate vaccines of various platforms and their numbers under clinical phase 1, 2, or III: Phase-wise distribution along with the numbers in the platform used are marked in the bar graph. **(D)** Summary of various vaccines’ clinical trial status is presented in the graph: Vaccine production from inception to commercialization is a prolonged process that requires multiple clinical trials, some of which have failed due to adverse effects.

In response to the outbreak, rapid diagnostics, speedy therapy, and vaccine research and development (R&D) are critical to curb the pandemic and prevent new viral outbreaks (Liu et al., 2020; Goel et al., 2021; Khan et al., 2021). Many attempts have been carried out on various platforms to develop a vaccine against SARS-CoV-2 (Callaway, 2020). Around 284 different candidate vaccines against COVID-19 are in development and are in the race to be introduced in the market after their successful phase III trials with prophylactic and safety data (WHO, 2021a). Due to the great impact on the lives of people and the economy from COVID-19, various vaccines got emergency approval after or before phase III trials (**Tables 1**, **2**). Currently, various platforms, e.g., nucleic acid (RNA/DNA), attenuated live, protein subunit, viral vector, whole virus inactivated, and VLP-based vaccines are being used to develop a safe and prophylactic vaccine against SARS-CoV-2 (**Figures 2A–G**). The vaccines by Pfizer, Moderna, Oxford/AstraZeneca (Vaxzevria/Covishield), Bharat Biotech (Covaxin), Gamaleya (sputnik V), Sinopharm, and Sinovac are in the market (**Table 2**) (Bos et al., 2020; Logunov et al., 2020a; Logunov et al., 2020b; Baden et al., 2021; Pfizer-Biontech, 2021; Voysey et al., 2021). However, detailed phase III clinical trial–based vaccine efficacy and safety data remain elusive for some approved vaccines and many candidates that are now under clinical trials (**Figure 3** and **Table 2**).

**Table 1:** Platform of COVID-19 Vaccine.

Platform	Type of candidate vaccine	Developer	Clinical phase trial
**DNA**	INO-4800+electroporation	Inovio Pharmaceuticals + International Vaccine Institute	NCT04642638 Phase II/III
	AG0301-COVID19	AnGes + Takara Bio + Osaka University	NCT04655625 Phase II/III
	nCov vaccine	Zydus Cadila	CTRI/2020/07/026352 Phase III
**RNA**	ARCT-021	Arcturus Therapeutics	NCT04668339 Phase II
	SARS-CoV-2 mRNA vaccine (ARCoV)	Academy of Military Science (AMS), Walvax Biotechnology and Suzhou Abogen Biosciences	NCT04847102 Phase III
	mRNA-1273.351. A lipid nanoparticle (LNP)-encapsulated mRNA-based vaccine that encodes for a full-length, prefusion stabilized S protein of the SARS-CoV-2 B.1.351 variant.	Moderna + National Institute of Allergy and Infectious Diseases (NIAID)	EUCTR2021-000930-32 Phase II
	CVnCoV Vaccine	CureVac AG	NCT04674189 Phase III
**Protein Subunit**	Recombinant SARS-CoV-2 vaccine (CHO Cell)	Anhui Zhifei Longcom Biopharmaceutical + Institute of Microbiology, Chinese Academy of Sciences	ChiCTR2000040153(Phase III
	VAT00002: SARS-CoV-2 S protein with adjuvant	Sanofi Pasteur + GSK	PACTR202011523101903 Phase III
	FINLAY-FR-2 anti-SARS-CoV-2 Vaccine (RBD chemically conjugated to tetanus toxoid plus adjuvant)	Instituto Finlay de Vacunas	RPCEC00000354 Phase III
	CIGB-66 (RBD+aluminum hydroxide)	Center for Genetic Engineering and Biotechnology (CIGB)	RPCEC00000359 Phase III
**Non-Replicating Viral Vector**	GRAd-COV2 (Replication defective Simian Adenovirus (GRAd) encoding S)	ReiThera + Leukocare + Univercells	NCT04791423 Phase II/III
**Replicating Viral Vector**	DelNS1-2019-nCoV-RBD-OPT1 (Intranasal flu-based-RBD)	University of Hong Kong, Xiamen University and Beijing Wantai Biological Pharmacy	Phase II ChiCTR2000039715
**Inactivated**	SARS-CoV-2 vaccine (vero cells)	Institute of Medical Biology + Chinese Academy of Medical Sciences	NCT04659239 Phase III
	QazCovid-in - COVID-19 inactivated vaccine	Research Institute for Biological Safety Problems, Rep of Kazakhstan	NCT04691908 Phase III
	Inactivated SARS-CoV-2 vaccine (Vero cell)	Beijing Minhai Biotechnology Co	NCT04852705 Phase III
	VLA2001	Valneva, National Institute for Health Research, United Kingdom	NCT04864561 Phase III
	COVID-19 inactivated vaccine	Shifa Pharmed Industrial Co	IRCT20201202049567N3 Phase II/III
**VLP**	Coronavirus-Like Particle COVID-19 (CoVLP)	Medicago Inc.	NCT04636697 Phase II/III

Platforms being used in development of vaccine against SARS CoV-2; these candidate vaccines are in either preclinical or various clinical phase trial stages and have not yet been licensed or launched by firms (WHO, 2021a).

**Table 2:** List of marketed vaccines against SARS-CoV-2.

Developer	Vaccine Name	Vaccine Type	SARS-CoV-2 Antigen	Doses and Route	Storage	Efficacy	Status	References
**Pfizer/BioNtech**	Comirnaty, tozinameran, BNT162b1 BNT162b2	mRNA encapsulated in lipid nanoparticle (LNP)	SARS-CoV-2 RBD and S protein full length in prefusion conformation for BNT162b1 and BNT162b2 respectively	Twice (3 weeks apart), I.M.	-70°C to -80°C (6 months)	95%	Emergency use in U.S., E.U. etc., Approved in several countries	(Polack et al., 2020)
**Moderna/NIAID**	mRNA-1273	mRNA encapsulated in lipid nanoparticle (LNP)	Full length S protein with prefusion conformation	Twice (4 weeks apart), I.M.	4°C (30 days), –20°C (6 months)	94.5%	Approved in Switzerland. Emergency use in U.S., U.K., E.U., others	(Voysey et al., 2021)
**Oxford University/AstraZeneca**	AZD1222/ChAdOx1 nCoV-19 (Covishield in India)	Attenuated adenoviral vector (nonreplicating) from chimpanzee ChAd	full-length codon-optimized S protein	Twice (4 weeks apart), I.M.	2°C–8°C	70%	Emergency use in U.K., E.U., India and other countries	(Voysey et al., 2021)
**Bharat Biotech/ICMR**	Covaxin, BBV152 A, B, C	Whole virion Inactivated SARS-CoV-2 vaccine + adjuvant	Whole virus	Twice (2 weeks apart), I.M.	2°C–8°C	81%	Emergency use in India	(Bharat Biotech, 2021a)
**Gamaleya**	Sputnik V, Gam-Covid-Vac	two nonreplicating viral vectors, adenovirus type 5 (rAd5) and adenovirus type 26 (rAd26)	SARS-CoV-2 full-length glycoprotein S	Twice (3 weeks apart), I.M.	-20°C	92%	Early use in Russia. Emergency use in other countries including India	(BBC News, 2020b)
**Beijing Institute of Biological Products/Sinopharm**	BBIBP-CorV	Inactivated SARS-CoV-2 vaccine (Vero cell)	Whole virus	Twice (3 weeks apart), I.M.	2°C–8°C	79%	Approved in China, U.A.E., Bahrain. Emergency use in Egypt, other countries	(BBC News, 2020a)
**Sinovac**	CoronaVac, PiCoVacc	Inactivated virus	Whole virus	Twice (2 weeks apart), I.M.	2°C–8°C	50%–91%	Approved in China. Emergency use in Brazil, Singapore, Malaysia, and Philippines	(BCB News, 2021)
**CanSino/BIB**	Convidecia, Ad5-nCoV	Adenovirus based viral vector (Ad5)- Nonreplicating	SARS-CoV-2 full length S protein	Single, I.M.	2°C–8°C	79%	Emergency use in China and Mexico	(BBC News, 2020a)
**Johnson & Johnson**	Ad26.COV2. S	Adenovirus based viral vector (Ad26)- Nonreplicating	SARS-CoV-2 full length S protein	Single, I.M.	-20°C (2 years), 2°C–8°C (3 months)	76.7%–85.4%	Applied for emergency use authorization in U.S.	(US FDA, 2021)
**Novavax**	NVX-CoV2373	S Protein adjuvanted with recombinant novavax protein	Trimeric SARS-CoV-2 full length S protein	Twice (3 weeks apart), I.M.	2°C–8°C (3 months), -20°C (2 years)	89.3% (P-3, UK), 90% (South Africa Trial)	Early use in UK and Australia	(Press release, 2021)
**Vector Institute**	EpiVacCorona	Chemically synthesized peptide antigens of SARS-CoV-2 proteins	Peptide Subunit Vaccine	Twice (3 weeks apart), I.M.	2°C–8°C (2 years)	100%	Early use in Russia	(Belta News, 2021)
**WIBP/Sinopharm**	WIBP-CorV	Inactivated virus propagated in Vero cells	Whole virus	Twice (3 weeks apart), I.M.	2°C–8°C	72.5%	Bahrain, Jordan, Egypt, UAE	(Reuters-Staff, 2021)

Vaccines developed by several companies and institutions on multiple platforms have been introduced in most countries. Vaccine efficacy is influenced by a variety of factors, including vaccine type, proper packaging, number of dosages, and booster requirements after a certain time interval as well as the route of administration. Vaccines with the potential to boost ongoing pandemic response have been introduced in many countries, on either an approved or emergency basis (Chakraborty et al., 2021; WHO, 2021b; Yan et al., 2021).

ICMR., Indian Council of Medical Research; WIBP., Wuhan Institute of Biological Products; BIB., Beijing Institute of Biotechnology; NIAID., National Institute of Allergy and Infectious Diseases; U.S., United State, EU., European Union; U.A.E., United Arab Emirates; U.K., United Kingdom; I.M., Intramuscular, P-3 (Phase-III trial).

**Figure 4:**
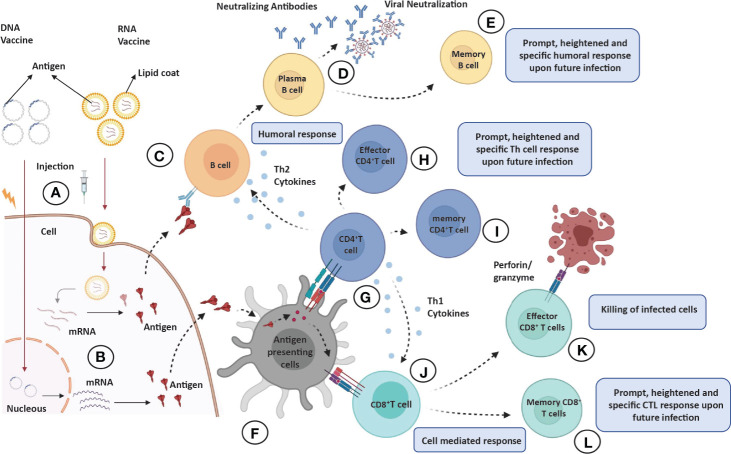
Overview of immune response elicited by various vaccine candidates. When administered into skin or muscle cells **(A)**, the nucleic acid expresses and codes for an immunogenic protein that mimics viral infection **(B)**. **(C–E)** Humoral responses: Antigens produced by skin/muscle cells are released in blood to activate the antibody response; antigen recognition by naïve B cells **(C)** leading to clonal selection and plasma cell (antibody secreting B cells) formation **(D)** and eventually production of long-lasting memory B cells **(E)**. **(F)** Antigen processing and presentation: The antigen produced by skin/muscle cells are captured by antigen-presenting cells (APCs; dendritic cells or macrophages) for processing and presented by MHC-II or MHC-I molecules on their surface. **(G–I)** CD4^+^T helper cells effector and memory response: The presented MHC-II molecule and antigen complex on APCs recognized by TCR and CD4 molecules on CD4^+^ Th cells **(G)** leading to production of effector CD4^+^ Th cells **(H)**, which produce sufficient levels of cytokines (Th2 cells produce TH2 cytokines IL-4 or IL-10 for humoral response and Th1 cells produce IL-12, IFN-Y for CTL response), and eventually, long-lasting memory CD4^+^ cells are generated **(I)**. **(J–L)** CD8^+^T helper cells effector and memory response: The presented antigen and MHC-II molecule complex on APCs recognized by TCR and CD8 molecules on CD8^+^ T cells **(J)** leading to production of effector CD8^+^T cells also known as cytotoxic T lymphocytes (CTL), which are responsible for killing the infected or self-altered cells **(K)**, and finally, long-lasting memory CD8^+^T cells are generated **(L)**. All memory cells provide long-lasting, heightened, and antigen-specific responses upon future infections.

To instigate the immune response, the protein antigens are achieved either by direct injecting protein subunit vaccines or by expression of genetic material delivered through viral or nonviral vectors into muscle cells (**Figures 4A, B**). Apart from antigens, the vaccine preparations also contain low toxic preservatives, such as 2-phenoxyethanol, to prevent vaccine contamination. The stabilizers, e.g., sugars (lactose, sucrose), amino acids (glycine), gelatin, or proteins, are used to prevent chemical reactions to achieve vaccine stability. Surfactants prevent clumping to keep the vaccine components homogeneous, and diluents (mostly distilled water) are used to achieve desired antigen concentration. Aluminum salts, such as aluminum phosphate, aluminum hydroxide, or potassium aluminum sulfate could be used as an adjuvant to enhance immune response. Vaccines may also contain traces of compounds used during vaccine manufacturing (https://www.who.int/news-room/feature-stories/detail/how-are-vaccines-developed).

**Figure 5:**
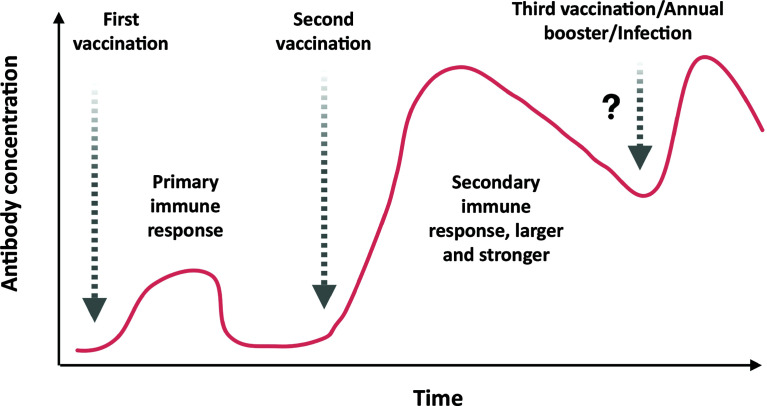
Antibody response to first, second, and probable third/annual booster dose of COVID-19 vaccination or infection: Initially, just after the first immunization, the vaccine does not elicit sufficient neutralizing antibody to prevent the infection of SARS-CoV-2. Upon administration of the second booster dose of vaccine, the vaccine elicits a stronger neutralizing antibody response with high titer that could prevent the infection efficiently. In some cases, third or annual boosters are required to revive the immune response against the pathogen.

Antigens in the circulatory system are recognized by antibodies on B cells, leading to isotype switching and antibody secreting plasma cell formation in germinal centers of secondary lymphoid organs. Some of the antibodies are capable of neutralizing the virus (neutralizing antibodies), which is crucial for clinical outcome (**Figures 4C, D**). Eventually, memory B cells are generated to withstand the onslaught of future infections (**Figure 4E**). The T cells, on the other hand, recognize antigens presented on (major histocompatibility complex) MHC-I or MHC-II of antigen-presenting cells (APCs) to activate CD8^+^T or CD4^+^T cells, respectively (**Figure 4F**). The activated helper T (effector CD4^+^T) cells secrete Th1 or Th2 cytokines for cell-mediated or humoral immune response against the pathogen (**Figures 4G, H**). However, the activated CD8^+^T cells develop into cytotoxic T cells responsible for the killing of infected cells (**Figures 4J, K**). Both CD4^+^ and CD8^+^ T cells develop a central memory response (**Figures 4I, L**). Most immunizations require booster doses to strengthen the immunological response induced by vaccines (**Figure 5**). A recent study suggests that S, M, and N proteins were strongly and nsp3, nsp4, and ORF8 were partially able to activate CD4^+^ T cells. On the other hand, the SARS-CoV-2 M and S proteins were strongly recognized by CD8^+^ T cells, and other antigens, such as nsp6, ORF3a, and the N protein, also showed significant reactivity (Grifoni et al., 2020).

The Wuhan strain of SARS-CoV-2 has undergone various mutations, resulting in B.1.1.7/alpha (United Kingdom), B.1.351/beta (South Africa), P.1/gamma (Brazil), B.1.617/delta (India), B.1.429 and B.1.427/epsilon (United States/California), B.1.525/eta (United Kingdom, Nigeria), P3/theta (Philippines), B.1.526/lota (United States/New York), B.1.617.1/kappa (India) and C.37/lambda (Peru) lineages (https://www.gisaid.org/hcov19-variants/). The complexity in the immune response elicited during COVID-19 infection and the probability of genomic changes render a huge challenge for the scientific fraternity regarding vaccines in the market and those under development. Cutting-edge strategy needs to be adapted to achieve a prophylactic vaccine against this dreadful virus. To achieve the aim of limiting the pandemic, we address the various vaccine types, their components, immune responses against various strains, hurdles, and future challenges.

## 2. Strategies Utilized in COVID-19 Vaccine Development

There are various strategies being utilized worldwide for vaccine development. An example of some of the forms of SARS-CoV-2 vaccine candidates and concepts are presented in **Figure 2**. Apart from emergency authorized vaccines for COVID-19, there are 100 candidate vaccines in the clinical evaluation phase, and 184 vaccines are in the preclinical evaluation stage (WHO, 2021a). These vaccines are being developed in various countries around the globe, including the United States, Germany, Austria, the United Kingdom, China, Australia, France, India, and Hong Kong (WHO, 2020). Development of efficacious and safe vaccines is urgently needed to curb the current pandemic. Vaccine developers must guarantee that people of all age groups, including with comorbidities, such as asthma and diabetes, can receive the COVID-19 vaccine as these categories of patients are at particularly high risk. It is, therefore, important to determine the level of safety provided by the vaccines, and patients may require more than one dose of the vaccine to preserve continued immunity to the virus. The production of a vaccine against SARS-CoV-2 is a challenging task due to many problems faced during the design phase. A variety of methods, including next-generation and traditional approaches, are being used to develop the vaccines, each one with different advantages and disadvantages (**Table 2**). The basic mechanism and details of immunological responses induced by different vaccines are shown in detail in **Figure 4**. Also, the predicted antibody response to first, second, and probable booster doses of the COVID-19 vaccination are represented in **Figure 5**. Live attenuated coronavirus vaccines provide the best protection, but their clearance is hampered by biosafety concerns. In contrast, inactivated coronavirus vaccines perform well in primate models and up to preclinical levels (Lundstrom, 2020). Because the immune systems of elderly people vary from those of healthy middle-aged adults, they sometimes do not respond equally well to immunization. Overall, there is no such thing as “one size fits all.” In general, we hope that adjuvants can aid ongoing vaccination campaigns across the world. Adjuvants are important for eliciting a stronger, long-lasting, and broader immune response, especially in people with compromised immune systems. **Figures 3A–C** shows the number of coronavirus vaccines in the preclinical and clinical stages based on the various platforms accessible and in a phase-by-phase approach. **Figure 3D** summarizes the coronavirus vaccine and the time it takes from development and preclinical testing, need to pass through phase 1, 2, and 3 trials. Few vaccines have been dropped because of their inability to induce a robust immune response, owing to negative side effects.

### 2.1. Inactivated Vaccine

Various vaccines for viral and bacterial diseases, including pertussis, rabies, hepatitis A, influenza, polio, and Japanese encephalitis, are based on an inactivated form of the vaccines. Usually, they do not provide protection as strong as live vaccines and require booster shots. Sixteen inactivated vaccines of SARS-CoV-2 are in advanced clinical trials. Bharat Biotech Covaxin (BBV152) is an inactivated COVID-19 vaccine being used in India. These vaccines require a BSL-3 facility for large-scale virus propagation, rendering this platform the most time-consuming and difficult. However, the benefit of using this platform is a multiple antigen-based vaccine that remains helpful in any viral mutation on a single protein. These inactivated vaccines lead to a strong neutralizing antibody response and give a more potent CD4^+^ T_h_1 cell response as compared with CD8^+^ T cells.

### 2.2. Live Attenuated Vaccine

Live vaccines are made from attenuated or weakened forms of the pathogen to stimulate a strong and long-lasting neutralizing antibody response along with both CD4^+^ T helper and CD8^+^ T cell mediated immunity by mimicking natural infection. However, extensive safety studies are required, and it could not be given to immunocompromised individuals. Currently, live attenuated vaccines are being used for rotavirus; chickenpox; yellow fever; and measles, mumps, and rubella. There are several benefits of a live-attenuated vaccine, including mounting an immune response to multiple virus antigens and the capacity to scale for mass production. The COVI-VAC, currently in clinical phase I trial, is a single-dose, intranasal, live-attenuated vaccine generated using Codagenix’s proprietary deoptimization technology in collaboration with the Serum Institute of India.

### 2.3. Viral Vector

Viral vector vaccines make use of a modified version of the pathogenic virus as a vector to provide immunity. Scientists all over the world are attempting to produce a COVID-19 vaccine dependent on replicating viral vectors, such as damaged measles, while others are focusing on nonreplicating viral vectors, such as adenovirus.

#### 2.3.1. Replicating

Three candidates from the replicating viral vector platform and two based on replicating viral vectors along with artificial APCs have reached the clinical phase. When cells are infected with the replicating viral vector vaccine, it not only produces a vaccine antigen, but it also produces more viral particles that can infect another cell. DelNS1-2019-nCoV-RBD-OPT1 is an intranasal spray vaccine based on the H1N1 influenza A virus. The dose is given twice, 28 days apart. It is currently being evaluated in a phase II clinical trial. A vaccine candidate developed by the Israel Institute for Biomedical Research makes use of recombinant vesicular stomatitis virus, in which the VSV-G protein is replaced with the SARS-CoV-2 S protein, creating a recombinant replicating virus. The candidate has reached phase I/II clinical trials for safety and efficacy evaluation.

#### 2.3.2. Nonreplicating

Nonreplicating viral vaccines are not able to produce new viral particles and are only involved in production of the vaccine antigen. Fifteen nonreplicating viral vector vaccines and one with an artificial APC is being evaluated in human clinical trials. CanSino Biologics and the Beijing Biotechnology Institute developed the novel coronavirus vaccine (adenovirus type 5 vector) candidate (Ad5-nCoV), and it has now been authorized and made accessible as a marketed vaccine. The vaccine candidate is based on the adenovirus-based viral vector vaccine technology framework of CanSinoBIO, which was previously used to successfully produce the globally groundbreaking Ebola virus infection vaccine (Zhao et al., 2020b). The neutralizing antibody response mediated by such vaccine platforms is determined by preexisting anti-vector immunity with enhanced CD4^+^ Th1 cell response and potent CD8^+^ T cell response.

### 2.4. DNA-Based Vaccine

The DNA-based vaccine, also known as the third-generation vaccine, contains DNA encoding specific proteins or antigens of the pathogen. Ten DNA vaccine candidates are in human clinical trials, and 16 are in preclinical trials. The proteins encoded by the DNA are translated into host cells, which are identified as a foreign material by the host immune system, thus inducing an immune response. INOVIA Pharmaceuticals, a biotechnology company, is currently focused on developing a DNA vaccine, which is in phase 2/3 clinical testing in the United States for COVID-19. Using INOVIO’s patented DNA medicine platform, INO-4800 was developed quickly after the genetic sequence of the coronavirus causing COVID-19 was released. A DNA-based vaccine that can be administered by a nasal spray is being produced by researchers at the University of Waterloo, Ontario, Canada. The vaccine operates using engineered bacteriophage, a process that enables the vaccine to activate the immune response in the lower respiratory tract of the nasal cavity and target tissues (WaterlooNews, 2020). DNA-based vaccines provide a sufficient neutralizing antibody response along with the CD4^+^ Th1 cell response. However, the CD8^+^ T cell response is less robust (Prompetchara et al., 2021).

### 2.5. RNA-Based Formulation

New technology is based on RNA, by which an mRNA-encoding pathogen’s proteins or antigens are used to elicit an immune response. Sixteen vaccine candidates based on mRNA formulation are being tested in the clinical phase. The mRNA-1273 is a novel lipid nanoparticle (LNP)-encapsulated mRNA-based vaccine that encodes for a full-length, prefusion stabilized spike (S) protein of SARS-CoV-2. Candidates for the vaccine include nucleoside modified mRNA (modRNA), mRNA-containing uridine (uRNA), and self-amplified mRNA (saRNA). An LNP formulation is combined with any mRNA format. These vaccine candidates for COVID-19 contain either the larger spike sequence or the smaller optimized RBD of the spike protein. The vaccines based on mRNA give an adequate neutralizing antibody response although the Th1 and Th2 cell response depends on the adjuvant used.

### 2.6. Protein Subunit Vaccine

A protein subunit vaccine uses protein fragments of the pathogen to elicit an immune response instead of introducing the whole pathogen. Currently, it is being used for hepatitis B, human papilloma virus (HPV), whooping cough, pneumococcal disease, meningococcal disease, and shingles. These vaccines provide a strong immune response against a particular antigen of the pathogen and are safe for all recipients, including individuals with a weakened immune system. Thirty-one such candidates are being assessed in human clinical trials before being available to the general population. Although some researchers want to inject coronavirus proteins directly into the bloodstream, it is also conceivable to employ protein fragments or protein shells that look like the coronavirus’s outer coat. Scientists and researchers are developing vaccines for viral protein subunits, the majority of which focus on the virus’s spike protein or a central portion known as the RBD. ExpreS2ion, a biotech company, uses its clinically validated Drosophila S2 insect cell expression system, ExpreS2, to produce SARS-CoV-2 viral antigens in the clinically validated cell lines. The goal is to generate and test vaccine antigens in mice to demonstrate immunogenicity and efficacy *in vitro*. The vaccine is in preclinical trials now. Protein subunit vaccines are well recognized for eliciting a strong neutralizing antibody response; however, the Th1 and Th2 cell response is influenced by the adjuvant employed.

### 2.7. Virus-Like Particle Vaccine

Virus-like particles (VLPs) are protein-based vaccines that stimulate high immune responses due to VLPs’ repetitive structures. These molecules mimic viruses but do not contain any viral genetic material and are not infectious. They are a very effective way of creating vaccines against diseases such as HPV, hepatitis B, malaria, and many more. There are five VLP-based vaccine candidates currently being evaluated in human clinical trials against SARS-CoV-2. The RBD SARS-CoV-2 HBsAg VLP vaccine is a subunit vaccine that uses RBD in a SARS-CoV-2 S protein conjugated with the hepatitis B surface antigen to stimulate the immune system to produce the anti-RBD antibody. The Central Committee on Research Involving Human Subjects in the Netherlands has approved an ABNCoV2 capsid virus-like particle–based COVID-19 vaccine developed by AdaptVac, a PREVENT-nCoV consortium member. The candidate vaccine is in phase I/II of study.

## 3. Approved COVID-19 Vaccines

Several COVID-19 vaccines have shown greater than 90% efficacy in preventing COVID-19 infections. A handful of vaccinations are presently authorized by both the national regulatory body and WHO. Some vaccines are in the process of being approved by WHO (WHO, 2021c).

### 3.1. Pfizer-BioNTech (BNT162b1 and BNT162b2)

The first time mRNA has been used as a vaccine platform was initially established by the Weissman group to develop a zika virus vaccine (Pardi et al., 2017). Pfizer-BioNTech developed BNT162b1 and BNT162b2 vaccines against SARS-CoV-2. BNT162b1 is a SARS-CoV-2 spike protein RBD encoding modified messenger RNA (mRNA) incorporating 1-methyl-pseudourine, which dampens innate immune sensing to induce mRNA translation. The RBD antigen is modified by the addition of a T4 fibritin-derived “foldon” trimerization domain to increase its immunogenicity by multivalent display (Mulligan et al., 2020). The other candidate, BNT162b2, encodes the full-length SARS-CoV-2 spike protein with two proline changes that lock it in the prefusion conformation and make it more closely mimic the intact virus to elicit prominent virus-neutralizing antibodies. Apart from mRNA (30 μg), the Pfizer-BioNTech vaccine includes lipids and cholesterol, potassium chloride, monobasic potassium phosphate, sodium chloride, dibasic sodium phosphate dihydrate, and sucrose. Storage is -70°C to -80°C; later, as an alternative, -25°C to -15°C was advised for 2 weeks (Polack et al., 2020). The European Medicines Agency (EMA) as a National Regulatory Authority (NRA) approved it, and later it was approved by WHO after evaluating the data on clinical trials (www.pfizer.com).

### 3.2. Moderna (mRNA1273)

The Moderna COVID-19 (mRNA1273) vaccine is a sterile, preservative-free, frozen suspension for intramuscular injection comprising the following ingredients: mRNA, lipids (SM-102, 1,2-dimyristoyl-rac-glycero tromethamine, tromethamine hydrochloride, acetic acid, sodium acetate, and sucrose), PEG2000-DMG, cholesterol, and 1,2-distearoyl-snglycero-3-phosphocholine [DSPC] (Moderna, 2021) (www.modernatx.com). The Moderna vaccine currently is licensed by EMA as well as WHO based on experimental evidence. It is a SARS-CoV-2 glycoprotein S-2P with a transmembrane anchor and an intact S1-S2 cleavage site, antigen-encoding, mRNA-based vaccine. The S2-P is stabilized in its prefusion conformation by 986 and 987 position proline substitutions at the top of the central helix in the S2 subunit. The lipid nanoparticle capsule, composed of four lipids, was formulated in a fixed ratio of mRNA and lipid. The dose is given intramuscularly in two doses 4 weeks apart. It can be stored in refrigerators for 30 days and at -20°C for 6 months. Primary efficacy analysis of the phase-III COVE study of mRNA-1273 involved 30,000 participants, including 196 cases of COVID-19, of which 30 cases were severe. Vaccine efficacy against COVID-19 was 94.1%, and vaccine efficacy against severe COVID-19 was 100%. mRNA-1273 continues to be generally well tolerated with no serious safety concerns identified to date (Baden et al., 2020).

### 3.3. Covaxin

Bharat Biotech’s Covaxin gained certification from the Drugs Controller General of India as an NRA, and it is currently under consideration by WHO for possible approval. This vaccine is developed by Bharat Biotech, India, using a coronavirus sample isolated from an asymptomatic patient (strain: NIV-2020-770) by the National Institute of Virology, India (Bharat Biotech, 2021a). It falls under the inactivated whole virion vaccine category that uses adjuvant Alhydroxiquim-II to boost immune response for long-lasting immunity. It contains 6 µg of whole-virion inactivated SARS-CoV-2 antigen and 250 µg aluminum hydroxide gel, 15 µg TLR 7/8 agonist (imidazoquinolinone), 2.5 mg TM 2-phenoxyethanol, and phosphate buffered saline up to 0.5 ml (https://www.bharatbiotech.com). The phase I and II clinical trials were conducted on 800 subjects, and the results demonstrated that the vaccine is safe and induced a robust immune response. The phase III study enrolled 25,800 participants between 18 and 98 years of age, including 10% over the age of 60, with analysis conducted 14 days post second dose. According to phase III analysis of Covaxin on 127 symptomatic cases, the vaccine showed a point estimate of 81% vaccine efficacy against mild and moderate COVID-19 disease. The efficacy against severe COVID-19 disease was 100% with an impact on reduction in hospitalizations. The efficacy against asymptomatic COVID-19 infection was 70%, suggesting decreased transmission in Covaxin recipients (Bharat Biotech, 2021b).

### 3.4. Oxford-AstraZeneca (AZD1222)

The chimpanzee adenovirus vectored vaccine containing the gene for expressing spike protein ChAdOx1-nCoV-19 (AZD1222) is developed by Oxford-AstraZeneca and manufactured by the Serum Institute of India. The vaccine is administered in two separate doses of 0.5 ml each given between 4 and 12 weeks apart and can be stored at temperatures 2°C to 8°C. In India, this vaccine is named Covishield, and it includes L-histidine, L-histidine hydrochloride monohydrate, polysorbate 80, ethanol, sucrose, sodium chloride, disodium edetate dihydride (EDTA), and water for infection. Initially the booster dose was given 4 weeks postimmunization; however, due to a shortage of the vaccine, published date sets allowed the booster dose to be administered 6 to 8 weeks apart. Further studies are required to adapt the best immunization policy for this vaccine. (www.astrazeneca.com). Analysis data from four ongoing blinded, randomized, controlled trials done across the United Kingdom, Brazil, and South Africa showed an overall efficacy of 70.4%; 11,636 participants (7548 in the United Kingdom, 4088 in Brazil) were included in the interim primary efficacy analysis. Participants aged 18 years and older were randomly assigned (1:1) to the ChAdOx1 nCoV-19 vaccine or the control (Voysey et al., 2021).

### 3.5. Sputnik V by Gamaleya (Gam-Covid-Vac)

Also known as Gam-Covid-Vac, this vaccine was developed by the Gamaleya Research Institute, part of Russia’s Ministry of Health. It is an adenoviral-based vaccine (dual viral vector) that uses a weakened virus, which, in turn, delivers small parts of the pathogen, ultimately stimulating an immune response. The gene encoding S-protein of SARS-CoV-2 is carried *via* an rAd26 vector (first dose) that provides humoral and cellular immunity, and the rAd5 vector (second dose) induces formation of memory cells. The active components are a modified replication-defective adenovirus of a different serotype modified to include the protein S-expressing gene of SARS-CoV-2. The ingredients include tris-(hydroxymethyl)-aminomethane, sodium chloride, sucrose, magnesium chloride hexahydrate, disodium EDTA dihydrate, polysorbate 80, ethanol, and water. The interim report of the phase III data include results for more than 20,000 participants aged 18 years and older, 75% of whom were assigned to receive the vaccine, and the follow up for adverse events and infection. No serious adverse events considered related to the vaccine were recorded, and vaccine efficacy, based on the numbers of confirmed COVID-19 cases from 21 days after the first dose of the vaccine, was reported as 92% (Logunov et al., 2021) (https://sputnikvaccine.com).

### 3.6. Sinovac (CoronaVac)

The CoronaVac vaccine was developed by Sinovac using inactive SARS-CoV-2 virus (CZ02 strain) along with aluminum hydroxide, disodium hydrogen phosphate dodecahydrate, disodium hydrogen phosphate monohydrate, and sodium chloride as adjuvant and stabilizing agents. Two doses of the vaccine about 14 to 28 days apart are administered. One main advantage associated with the vaccine developed by Sinovac is that it can be stored at 2°C–8°C. China has approved the Sinovac vaccine for emergency use since July 2020 through the National Medical Products Administration (NMPA), and later, it got approval by WHO. Other countries, such as Indonesia, Turkey, Brazil, and Chile, have also authorized emergency use of this vaccine (http://www.sinovac.com). The phase III trials conducted in Brazil and Turkey evaluated the efficacy of the vaccine candidate in healthcare workers who provide treatment to COVID-19 patients. Both trial studies were randomized, double-blind, and placebo controlled. There were 12,396 health workers over 18 years old enrolled. A total of 253 positive cases were collected during the observation period. After 14 days following vaccination with two doses of the vaccine following a 0-, 14-day schedule, the efficacy rate against diseases caused by COVID-19 was 50.65% for all cases; 83.70% for cases requiring medical treatment; and 100.00% for hospitalized, severe, and fatal cases (Palacios et al., 2020).

### 3.7. Sinopharm (BBIBP-CorV)

China has approved the Sinopharm multivariant vaccine (Sinopharm’s BBIBP-CorV), which is a WHO approved inactivated virus vaccine. Of the three coronavirus variants obtained from patients in China, the variant that multiplied the most quickly in monkey kidney cells was chosen and was inactivated using chemical beta-propiolactone. It was then treated with an aluminum-based adjuvant to increase its immunogenicity. Unlike the Moderna and Pfizer vaccines, this vaccine is easy to transport and can be stored at 2°C–8°C (BBC News, 2020a). Being multivariant, the immune response induced by the BBIBP-CorV vaccine could not be impacted by a mutation in the virus (http://www.sinopharm.com). According to a China National Biotec Group Company study, more than 40,000 people in the United Arab Emirates and Bahrain aged 18 and above without a known history of COVID-19 participated in the trials. The vaccine showed an efficacy rate of 79% against symptomatic COVID-19 cases with rare serious adverse effects reported (Al Kaabi et al., 2021).

### 3.8. CanSino (Convidecia)

The CanSino vaccine from China is approved by the NMPA, China, and is in the process of getting approval from WHO. The vaccine developed by CanSino, Ad5-nCoV, is a nonreplicating human adenoviral (Ad5)-based vaccine that expresses a full-length spike glycoprotein of coronavirus. It was originally tested in mice and ferrets and was the first vaccine to enter human trials in March 2020. The phase III trial NCT04540419 with 500 participants determined a single intramuscular shot of 5 × 10^10^ virus particles proved to be well tolerable and immunogenic (Logunov et al., 2020a). Mexico became the first country to give emergency use approval to this vaccine developed by CanSino Biologics and People’s Liberation Army scientists (http://www.cansinotech.com). CanSino Biologics conducted its phase III trials in Argentina, Chile, Mexico, Pakistan, Russia, and Saudi Arabia with 40,000 participants. The company announced that the interim analysis data of the phase III clinical trial of Convidecia shows that the vaccine candidate has an overall efficacy of 65.28% at preventing all symptomatic COVID-19 disease 28 days after single-dose vaccination and an efficacy of 90.07% at preventing severe disease 28 days after single-dose vaccination (https://www.precisionvaccinations.com/vaccines/convidicea-vaccine).

### 3.9. Janssen (Janssen COVID-19 Vaccine)

Janssen got licensed from EMA and approved by WHO. The vaccine developed by the Janssen and Prevention BV subsidiary of Johnson and Johnson, the Ad26.CoV.S, is based on human adenovirus Ad26 and expresses the full-length spike protein (Bos et al., 2020). The vaccine contains genetically modified organisms and ethanol derived from corn or vegetables (https://www.janssenmd.com). The vaccine can remain stable for two years at -20°C and a maximum of 3 months at temperatures of 2°C–8°C. The phase III trial was conducted across eight different countries. The U.S. Food and Drug Administration has issued emergency use authorization to this vaccine in individuals 18 years of age and older. In an international, randomized, double-blind, placebo-controlled, phase III trial, Johnson & Johnson randomly assigned adult participants in a 1:1 ratio to receive a single dose of Ad26.COV2.S (5×10^10^ viral particles) or placebo. In the per-protocol at-risk population, 468 centrally confirmed cases of symptomatic COVID-19 with an onset at least 14 days after administration were observed, of which 464 were moderate to severe–critical (116 cases in the vaccine group vs. 348 in the placebo group), which indicates a vaccine efficacy of 66.9% (Olliaro et al., 2021).

### 3.10. Novavax (NVX-CoV2373)

Novavax has been licensed by the EMA and is now being reviewed by WHO. The vaccine developed by Novavax is a protein subunit–based vaccine developed by using a SARS-CoV-2 spike protein subunit in its glycosylated form. The vaccine can be stored stably at 2°C–8°C and requires two doses 3 weeks apart. To increase the immune response, it is mixed with Novavax’s patented saponin-based Matrix-M adjuvant (https://www.novavax.com). In the 15,000-subject UK phase III clinical trial (NCT04583995), Novavax saw 56 cases of COVID-19 in the placebo arm and six cases in the NVX-CoV2373 group at the interim analysis, resulting in an overall efficacy of 89.3%. All the cases in the vaccine cohort were mild or moderate. Twenty-seven percent of subjects were aged over 65 years. The company said that 32 of the COVID-19 cases were infected with the B.1.1.7 variant. A *post hoc* analysis put the efficacy against B.1.1.7 at 85.6% compared with an efficacy of 95.6% versus older variants. The Novavax clinical trials represent the first major controlled clinical tests of how a COVID-19 vaccine performs against the B.1.1.7 and B.1.351 variants. Overall, the results suggest NVX-CoV2373 may be as effective as any prophylactic studied to date against older variants (Shinde et al., 2021).

### 3.11. Vector Institute (EpiVac Corona)

EpiVac corona got approval from the Russian NRA and currently is in the process to get approval by WHO. The EpiVac corona vaccine uses chemically synthesized peptide antigens of SARS-CoV-2 along with aluminum hydroxide as an adjuvant. The vaccine is administered intramuscularly twice 21–28 days apart (Belta News, 2021). The phase I and II studies tested the safety, side effects, and immunogenicity of the potential vaccine in 100 people aged 18–60, according to the state trials register. The immunological effectiveness of the EpiVac corona vaccine was found to be 100% (Ryzhikov A.B., 2021).

### 3.12. WIBP/Sinopharm (WIBP-CorV)

Another vaccine by Sinopharm uses a whole inactivated virus. The double-blind, randomized, phase III trial designed by the Wuhan Institute of Biological Products Co., Ltd., and the Beijing Institute of Biological Products Co., Ltd., on 3469 participants, including adults of 18 years and older age without prior known history of SARS-CoV, SARS-CoV-2, or Middle East respiratory syndrome infection (via onsite inquiry) were enrolled, showed an efficacy rate of 72.8% against symptomatic COVID-19 cases (Al Kaabi et al., 2021).

## 4. Challenges in Vaccine Design

**Figure 6:**
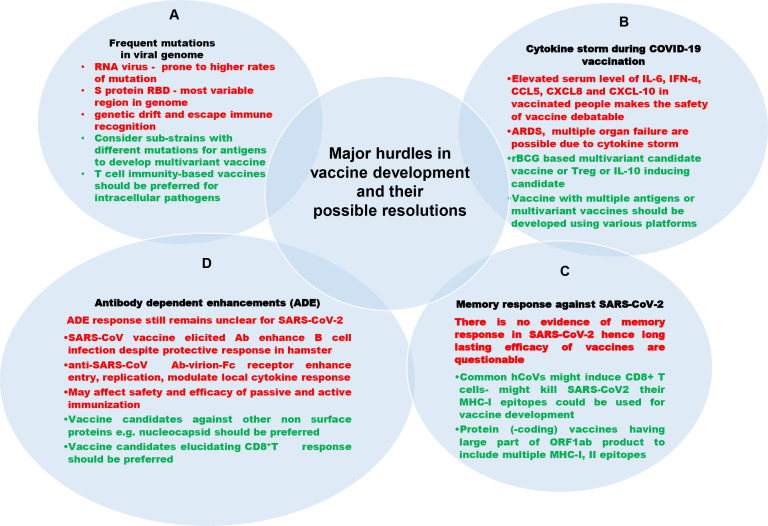
Challenges in developing vaccines against SARS-CoV-2 (black color text), their description (red color text) and their possible resolutions (green color text). **(A)** Mutation in viral genome: The RNA viruses including SARS-CoV-2 are more prone to mutation in their genome, leading to viral immune evasion. **(B)** Cytokine storm during COVID-19 vaccination: Occasionally, the vaccination may lead to cytokine storm and other complications raised by it. **(C)** Lack of memory response: Any vaccine provides long-lasting efficacy only because of memory responses of adaptive immunity. No vaccine against SARS-CoV-2 is documented to have established memory response postvaccination. The neutralizing antibody fades away a few months post vaccination. **(D)** Antibody-dependent enhancements: There is the possibility of an antibody-mediated increase in infection called antibody dependent enhancement.

Various precedented and unprecedented hurdles are encountered by designing and manufacturing agencies to develop an effective and prophylactic vaccine against novel viruses. Moreover, for a new vaccine candidate, it takes 10–15 years to be introduced for public use after rigorous and cumbersome clinical trials. Furthermore, most companies are unwilling to engage in vaccine production because vaccine profit margins are incompatible with those for drug development. Here, we discuss various challenges in SARS-CoV-2 vaccine development and propose possible solutions for respective problems (**Figure 6**).

### 4.1. Mutation

The novel coronavirus, like other RNA viruses, evolves by multiplying genetic material with higher rates of mutation (Raghuram et al., 2015; Islamuddin et al., 2018), resulting in greater genetic variation over time. Additionally, its capacity to shift genetic material from human to human and mutate in the human body allows it to be more transmissible (Denison et al., 2011). The rise of these variants may impact vaccine development and therapies as they spread throughout the population all over the world (**Table 3**) within a short span of time. Out of three types of possible mutations—base deletions, insertions, and substitutions—SARS-CoV-2 shows deletion and substitution mutations. Missense mutations account for 34.3%, nonsense mutations for 6.7%, and silent mutations for 0.8% of the SARS-CoV-2 genome (Pathan et al., 2020). Researchers found that a highly variable region of the spike protein in circulating viruses accounts for the top 10% of entropy (rate of mutation). A change in the amino acid in the antigenic part may hamper the neutralizing antibody functionality and could affect the degree of protection by vaccines. Importantly, to prevent the derailing of promising vaccines or antibody-based prophylactics or treatments, it is critical to understand how and whether SARS-CoV-2 may evolve to evade antibody-dependent immunity. Intriguingly, the most variable region in the coronavirus genome is the RBD of the spike protein (Zhou et al., 2020). Greaney et al. (2021b) have performed mutational scanning to detect the possible mutations in the RBD of spike protein that could affect neutralizing antibody functionality. It is, therefore, imperative to consider various clan, variants, and substrains with different mutations to reduce possibilities that will impact vaccine potential. The N439K mutation in RBD is the second most common mutation in RBD and the sixth most common mutation in the spike; it increases the binding affinity of spike with ACE-II twofold because of the salt bridge formed at the binding interface with a positively charged amino acid. This has been observed in more than 30 countries. The variant was first identified in March 2020 in Scotland, and as of January 2021, a second lineage B.1.258 has independently emerged in other European countries (**Table 3**). The N439K mutation did not change the clinical spectrum of the disease; however, in *in vivo* studies, the viral loads were increased compared with wild-type viruses with N439 residue. Also, D614G is one of the most prevalent nonsynonymous mutations of the spike protein that dominate the global pandemic (**Table 3**). The D614G mutation, like the N439K mutation, is shown to enhance viral infectivity *in vitro* and enhance viral fusion with the ACE2 receptor, but there has been no evidence of a connection between this mutation and disease severity (Hou et al., 2020; Zhang et al., 2020; Groves et al., 2021).

**Table 3:** Major SARS-CoV-2 variants.

Type	First detected in country	Variation in spike	Risk and impact on vaccine efficacy	References
VOC 202012/01 GRY (B.1.1.7)	U.K.	N501Y 144Y del 69/70 deletion P681H, D614G	Increased risk of death compared with other variants. ~50% increased transmission, Minimal impact on neutralization by convalescent and post-vaccination sera	(Arif, 2021; CDC, 2021b; Collier et al., 2021; Edara et al., 2021; Emary et al., 2021; Galloway et al., 2021; Muik et al., 2021; Ostrov, 2021; Planas et al., 2021; Shen et al., 2021; Wang et al., 2021a; Wu et al., 2021)
VOC GH/501Y.V2 (B.1.351)	South Africa	Shares some mutations with B.1.1.7 K417N, E484K N501Y, D614G	No evidence to suggest that this variant has any impact on disease severity or impact on neutralizing antibodies.	(Kim et al., 2021; Planas et al., 2021; Wang et al., 2021b; Zhou et al., 2021)
VOC GR/501Y.V3 (P.1)	Brazil, Japan	17 unique mutations, K417T, E484K, and N501Y	The emergence of this variant raises concerns of a potential increase in transmissibility or propensity for SARS-CoV-2 re-infection of individuals.	(CDC, 2021b; Dejnirattisai et al., 2021; Hoffmann et al., 2021)
VUI G/484K.V3 (B.1.525)	UK, Nigeria	E484K Q677H F888L	E484K mutation associated with potential immune escape.	(Jangra et al., 2021)
VOC G/452R.V3 (B.1.617+)	India	L452R, E484Q, D614G	Slightly reduced neutralization by post-vaccination sera	(Greaney et al., 2021a; Yadav et al., 2021)
VUI GH/452R.V1 (B.1.429)	USA	S13I, W152C, L452R, D614G	~20% increased transmissibility, Reduced neutralization by convalescent and postvaccination sera	(Deng et al., 2021)

Scientists are looking into how effective a vaccination might be in protecting persons infected with SARS-CoV-2 variants. The table displays the most common variants, countries first detected in, newly identified mutations in spike protein, and the risks they bring to public health (CDC, 2021a).

### 4.2. Global Variants of SARS-CoV-2

Various mutations, leading to rapidly spreading SARS-CoV-2 variants in the United Kingdom (B.1.1.7), South Africa (B.1.351), India (B.1.617), Brazil (P1), the United States (B1.429), and United Kingdom/Nigeria (B.1.525) strains, propagate even more rapidly, and this could contribute to more cases of COVID-19 (**Table 3**). It is obvious that a rise in the number of cases would put pressure on healthcare infrastructure and may lead to more fatalities. Current research indicates that antibodies produced by vaccines administered to indigenous people respond to these variants; however, more evidence and research is required to confirm this. In various studies, the mutations or their combinations showed diminished neutralizing activity of serum from mRNA-vaccinated people (Diamond et al., 2021; Zeyaullah et al., 2021).

In addition, there is always a possibility that these mutations may hamper the therapeutic antibody functions and, therefore, impact the efficacy of the vaccines. Furthermore, a human infected with one strain is more likely to become immune to another variant or strain, and if it failed to do so, then it clearly necessitates the development of new vaccination strategies to produce preventive vaccines that can combat the risk of many of these variants.

### 4.4. Antibody Dependent Enhancements (ADE)

Virus-specific antibodies control infections by neutralizing the virus. However, in ADE, the antibodies may be helpful to the virus by facilitating its entry into the cells through Fc receptors. Some vaccines against dengue and zika viruses are reported to have ADE effects (Khandia et al., 2018). Previously, it is shown that neutralizing antibodies against RBD of MERS-CoV and SARS-CoV facilitated virus entry into Fc receptor−expressing human cells *in vitro* (Wan et al., 2020). The incidence of SARS-CoV-2 ADE has not yet been proven (Wang et al., 2014; Negro, 2020; Wan et al., 2020). An alternative target to overcome ADE may be the nonsurface proteins, such as the nucleocapsid (N) protein. Because the N protein is not on the virus surface, its antibodies cannot promote virus entry. Despite defensive responses in the hamster model, antibodies elicited by the SARS-CoV vaccine strengthened infection of B cell lines (Li et al., 2018). On the other hand, anti-nucleocapsid antibodies neither neutralized infection nor induced ADE. Therefore, the interaction with Fc receptors of virion-complexed anti-SARS-CoV antibodies can result in both increased viral cell entry and replication and clinically impactful regulation of the local cytokine response. ADE infection has become a major concern for the prevention of diseases by vaccination. ADE response studies should be included for all candidate vaccines as a major safety parameter for approval of the vaccine.

### 4.5. Immune Evasion by Coronavirus

Studying the immune responses to SARS-CoV-2 and its approaches to immunoevasion enhance our understanding of pathogenesis and viral clearance and lead to the development and assessment of vaccines and immune therapeutics. The subsequent immune reactions, immunopathology, and processes of immune evasion are underevaluated. SARS-CoV-2 has developed various mechanisms to avoid innate immune detection like many other viruses, including low levels of cytosine-phosphate-guanosine (CpG) in the genome, glycosylation to shield the interacting receptor to the host, RNA shielding, and viral protein generation, that effectively hinder a virus. These mechanisms together allow efficient infection and increased viral load. Innate immune evasion is enabled by cap-methylation of viral RNA, and inhibition of steps in the IFN form I/III pathways are included in such evasion. SARS-CoV-2 has established the most severe CpG deficiency of all betacoronaviruses in accordance with other viruses (Xia, 2020), thus preventing ZAP action. The processing of capping the 5’ end is another technique for defending mRNA used by the host and several viruses. Capping restricts degradation and greatly inhibits identification by cytosolic pattern recognition receptors (PRRs) for both host and virus RNA. The post-translational modifications of structural and nonstructural proteins alter the target epitope, leading to compromised vaccine efficacy (Jeyanathan et al., 2020). Importantly, RNA viruses, including SARS-CoV-2, go through frequent mutation to have multiple variants that lead to immune evasion by viruses. This immune evasion makes it difficult to develop efficient vaccines, especially in the case of variants, but it allows the development of multiple epitope-based vaccine candidates, so in case one epitope does not respond, the other could take over and eventually enhance vaccine efficacy.

### 4.6. Cytokine Storm in COVID-19 Vaccination

Cytokine storm is a deadly, unregulated systemic inflammatory response resulting from the release of large amounts of pro-inflammatory cytokines (IFN-α, IFN-γ, IL-1β, IL-6, IL-12, IL-18, IL-33, TNF-α, etc.) and chemokines (CCL2, CCL3, CCL5, CXCL8, CXCL9, CXCL10, etc.) by immune effector cells in response to SARS-CoV-2 infection. The uncontrolled cytokine response is one of the main mechanisms for ARDS, responsible for mortality in COVID-19 patients (Williams and Chambers, 2014; Min et al., 2016). Similar ARDS-like immunopathology was observed in SARS-CoV and MERS-CoV (Williams and Chambers, 2014). Moreover, individuals with serious MERS-CoV infection display elevated serum levels of IL-6, IFN-α, and CCL-5, CXCL-8, CXCL-10 compared with those with mild-to-moderate disease, comparable to those with SARS-CoV (Coperchini et al., 2020). In extreme cases of SARS-CoV-2 infection, as is the case with SARS-CoV and MERS CoV infections, the cytokine storm forces the immune system to attack the body aggressively, causing ARDS, multiple organ failure, and eventually death. (Xu et al., 2020). The vaccines should be able to suppress cytokine storm or at least hamper its deleterious impact, e.g., the IL-10 and Th-2 cytokine could be helpful in controlling cytokine storm (Costela-Ruiz et al., 2020).

Most of the candidate vaccines for SARS-CoV-2 aim to boost the immune system as much as possible to induce strong immunization. This may lead to uncontrolled cytokine storm among some vaccinated individuals, which may lead to ARDS, one of the most common causes of death in COVID-19 patients. Vaccination in 33 African green monkeys and 200 mice induced protective immunity against SARS-Cov-2. Approximately 7% of monkeys along with 5% of experimental mice showed cytokine storm in their lungs upon post mRNA vaccination challenge by SARS-CoV-2. It seems developing a vaccine for SARS-Cov-2 that can regulate cytokine storm is needed (Farshi, 2020).

### 4.7. Memory Response Against SARS-CoV-2

The efficacy of a vaccine against the pathogen is confirmed by the memory response at the time of the second or subsequent attack. The reinfection in COVID-19 patients demonstrates a lack of suitable memory response after prior infection probably due to the viral immune evasion strategy. The candidate vaccines should be capable of eliciting B and T cell memory responses. Ironically, any approved vaccine does not have well-established memory response data, and it is imperative that it be deciphered for all candidate vaccines. There are some potential ways for this likely immune memory to be manipulated. For instance, it may be best to use SARS-CoV-2 genes that encode MHC-I epitopes that fit those of common coronaviruses when using RNA for immunization (Kupferschmidt and Cohen, 2020). For the most part, when considering potential strategies for SARS-CoV-2 vaccination, consideration should be given to preexisting MHC-I-based immunity resulting from previous common coronavirus infections. Common human coronaviruses can also induce some MHC-II-mediated immune memory by CD4^+^ helper T cells with regard to SARS-CoV-2 recognition for shared epitope use by various coronaviruses. CD4^+^ helper T cells can help stimulate cells involved in cytotoxic immune responses mediated by antibodies or cells. Recent studies also demonstrate that previously SARS-CoV infected patients show long-lasting SARS nucleoprotein (NP)-specific memory T cells displaying cross-reactivity to SARS-CoV-2 NP (Le Bert et al., 2020a). The promiscuous common T cell epitopes, which could elicit both CD4 and CD8^+^ T cells against SARS-CoV, SARS-CoV-2, and MERS, should be taken into consideration for vaccine development. For all vaccines, the studies should be conducted on vaccine recipient individual samples for detection of long-term protection, specifically memory T and B cells. Additionally, the virus neutralization assay should be performed to detect functional antibodies in the serum of vaccine recipient individuals.

### 4.8. Animal Model for Vaccine Study

Animals play a crucial role in the development of COVID-19 treatments and vaccines. The race to generate and expand mice for COVID-19 research is going on in the lab. The introduction of a safe and predictable COVID-19 animal model of infection is indeed a welcome development for preliminary antiviral and vaccine evaluations, and it is likely to help researchers understand the immune evasion mechanism adopted by various strains of the virus. The hamster model is not new to corona virology, but it has the ability to offer a more stable and predictable model of infection than murine models (Roberts et al., 2005). In the murine model, hyper-accentuated immune responses after SARS-CoV-2 vaccination have previously been published (Bolles et al., 2011). In addition, transgenic animals are often employed as models in biomedical research in the lab. Genetically engineered animals, mostly mice, account for almost 95% of those utilized. They are the tools for studying the disease susceptibility, progression, and treatment response. Following the discovery of ACE-2 as the SARS-CoV host receptor, there has been a lot of interest in establishing human disease–like mouse models. This resulted in the creation of transgenic mice with human hACE2, K18-hACE2, expressed in the epithelia of tissues and organs, such as the lungs, liver, kidney, spleen, heart, and intestine (Kumar et al., 2020). They are very susceptible to SARS-CoV infection with high viral lung titers, considerable weight loss, and morbidity. The ACE-2 mouse model becomes the tool set for cardiovascular and pulmonary research and is critical to advancing medicine and technology to search for drugs and a vaccine to fight the COVID-19 pandemic (Jia et al., 2020).

### 4.9. Storage, Transportation, and Handling of Vaccines

Most of the vaccines, especially the mRNA vaccines, as observed, require significantly low storage temperatures that are below the freezing point, which is difficult to maintain, particularly for longer durations, and poses a huge limitation in developing countries. The vaccines developed by Pfizer, Moderna, and Johnson & Johnson require storage of vaccine vials at -70°C to -80°C, -20°C (6 months), and -20°C (2 years), respectively, thus making them extremely fragile and difficult to store and handle, especially in developing countries. Apart from the requirement of ultralow freezing temperatures, the vaccines must be protected from direct exposure to sunlight and ultraviolet light, making the handling all that more difficult. The storage and transportation requirement puts extra financial burden on the vaccine recipient. Vaccines having room temperature storage are always cost effective and ease point of care units. The advantages and disadvantages of the major vaccine platforms are mentioned in **Table 4**.

**Table 4:** Advantages and disadvantages of the major vaccine platforms (Kyriakidis et al., 2021).

Vaccine Type	Advantages	Disadvantages
Nucleic acid vaccines	Scalability. Fast design and development. Extremely safe. No infectious agent handling is required. Can induce humoral and cellular responses.	Currently, few nucleic acid vaccines approved, including vaccine for SARS-CoV-2. DNA vaccines require a special delivery platform. mRNA vaccines exhibit instability and require storage at less than -20°C.
Viral-vectored vaccines^π^	Can induce robust humoral and cellular responses with a single dose. Good safety profile.	Preexisting immunity against a human viral vector can attenuate immune responses. Some candidates require storage at less than -20°C.
Protein Subunit vaccines^£^	Safety during production. Can be safely administered to immunosuppressed people. No infectious agent handling is required.	Small size of antigens diminishes their uptake by antigen-presenting cells (APCs). Low immunogenicity. Need several booster doses and adjuvants. Do not elicit cellular responses. Antigen integrity needs to be confirmed. Production limited by antigen production scalability.
Polysaccharide vaccines^¥^	Provides an alternative for vaccines against pathogens with an abundance of polysaccharide antigens (mostly bacteria).	Boost doses seldomly enhance the responses. Only IgM isotype and IgG2 subtype are induced, leading to limited antibody mediated effector functions. Poor memory responses. Works poorly on children.
Conjugate vaccines^≠^	Enhances the poor immunologic responses produced by polysaccharide vaccines as it induces T-dependent responses.	Absence of cellular responses. Adjuvant and booster doses needed.
Virus-like particles vaccines^±^	They combine the efficacy of attenuated vaccines and the safety of subunit vaccines. Scalability of production. Their size makes them ideal for uptake by APCs.	The assembly of the particles is sometimes challenging.

An example of vaccine used with this approach [^Π^Ebola; ^£^Hep B, Hep C, Influenza, Human Papilloma Virus (HPV); ^¥^Pneumococcal polysaccharide vaccine (PPSV or PPV-23); ^≠^Streptococcus pneumoniae vaccine, Neisseria meningitidis conjugated vaccine (meningococcal vaccine), Typhoid vaccine, Haemophilus influenzae type b vaccine; ^±^HPV, Hep B].

## 5. Possible Resolutions to Overcome Hurdles in Vaccine Development Against SARS CoV-2

### 5.1. Vaccines Focused on T Cell Immunity and Memory

Most of the vaccine candidates against SARS-CoV-2 are focused on developing neutralization antibodies against the viral spike protein to diminish viral entry into host cells. However, to develop a prophylactic vaccine against intracellular pathogen-like viruses, the cytotoxic T lymphocyte (CTL) response remains indispensable (Ahmed and Akondy, 2011). A group recently suggested that the severe infection of COVID-19 provides better memory T cells than milder infection (Chen and John Wherry, 2020). Earlier studies demonstrate that only 50% of SARS survivors showed viral-specific B and T cell responses (Cao et al., 2007; Tang et al., 2011; Ng et al., 2016; Le Bert et al., 2020b). However, T cell response was long-lasting (6–17 years), associated with less severe disease, cleared the virus rapidly, and did not evolve into an ADE response. On the other hand, B cell response was short-lived (3 years), associated with severe disease (MERS survivors with higher antibody titer must stay longer in ICU), did not clear the virus rapidly, and was found to be involved in ADE (Cao et al., 2007; Tang et al., 2011; Ng et al., 2016; Zhao et al., 2017; Liu et al., 2019; Le Bert et al., 2020b; Sekine et al., 2020; Lynch et al., 2021). A high serum level in SARS-CoV-2 infection is associated with longer stay of patients in the ICU and the need of ventilators because of severe disease condition (Cao, 2020; Zhao et al., 2020a). Thus, they may serve as candidates for designing SARS-CoV-2 vaccines. Another eight immunodominant CD4+ T cell epitopes have been suggested for use in a subunit vaccine to potentially elicit effective T and B cell responses. They are distributed across the S (232–246 and 233–247), E (55–69, 56–70, and 57–71), and M (97–111, 98–112, and 99–113) proteins. These predictions warrant further investigation and may aid in effective vaccine design against SARS-CoV-2. In addition to a strong antibody response, the coronavirus vaccines should also be capable of inducing virus-specific CD8+ T cell immunity. In addition, the focus should also be made on memory response of both humoral as well as cell-mediated responses.

### 5.2. Development of Multivariant Vaccines

Most viruses evolve and change over time, and SARS-CoV-2 is no exception with its composition constantly changing and raising concerns about the efficacy of existing COVID-19 vaccinations in use. With the appearance of three new strains, B.1.1.7 (United Kingdom), B.1.351 (South Africa), and P.1 (Brazil) as well as B.1.617 (India), there is a need to develop a multivariant vaccine (**Table 3**). Likewise, annual influenza vaccinations are administered to people in many countries every year. The constant mutation in the influenza virus yearly design of new vaccines is predicted on the basis of the current influenza viral strain. Both trivalent and quadrivalent vaccines targeting three and four strains of the virus, respectively, are used as flu vaccines (Rudenko et al., 2018). Gritstone Oncology, Inc., in Emeryville, California, with support from the Bill and Melinda Gates Foundation and the National Institute of Allergy and Infectious Diseases (NIAID), is working on a multivariant vaccine that targets epitopes on the spike protein as well as other areas of the virus for attack by T cells. It is an mRNA vaccine using adenovirus vectors for delivery. The Gates Foundation supported preclinical development of the vaccine, and NIAID has partnered with the company on an early stage clinical trial. Preclinical tests revealed that more regions of the SARS-CoV-2 virus elicited multiple immune responses, including high-titer neutralizing antibodies and CD8^+^ T cell responses against the spike protein as well as a broad CD8^+^ T cell response against epitopes from multiple viral genes beyond the spike. There are currently no approved multivariant vaccines, but efforts are underway to develop them in the future. We recommend that we proceed with the development of a SARS-CoV-2 vaccine by aiming to build a multivariant vaccine for improved protection.

### 5.3. BCG as a Platform for Live-Attenuated Vaccine

Recently, various reports suggest that countries with a non-BCG vaccine recipient population (Italy, Nederland, the United States) show higher case fatality rates compared with long-standing universal BCG policy-practicing countries (Hegarty et al., 2020; Miller et al., 2020). In addition, in the elderly population (Gursel and Gursel, 2020), BCG is suggestive of the notion that BCG protects the vaccinated elderly population. Its known protective immunological benefits, decreased incidences, hampered disease transmission and progression, and lowered mortality are suggestive of BCG vaccination as a potential nonspecific safe tool against COVID-19; however, various other factors make BCG efficacy against COVID-19 debatable (Dayal and Gupta, 2020; Gursel and Gursel, 2020; Hegarty et al., 2020; Hensel et al., 2020; Kirov, 2020; Miller et al., 2020).

#### 5.3.1. BCG Vaccination Renders Nonspecific and Variable Immune Response

BCG vaccination in healthy volunteers increases IFN-γ; enhances monocyte-derived cytokines TNF and IL-1β release; and elevates activation markers CD11b and PRRs such as Toll-like receptor-4, CD-14, and scavenger receptors (Kleinnijenhuis et al., 2012; Goswami et al., 2020). BCG vaccination in infants, surprisingly, increases 11 cytokines and chemokines in response to different nonspecific innate immunity stimuli, which includes epidermal growth factor, eotaxin, IL-6, IL-7, IL-8, IL-10, IL-12p40, monocyte chemotactic protein-3, macrophage inflammatory protein-1α, soluble CD-40 ligand, and platelet-derived growth factor. Moreover, in monocytes, the heterologous production of Th1 (IFN-γ) and Th17 (IL-17 and IL-22) immune responses to non-mycobacterial stimulation remained strongly elevated even 1 year after BCG vaccination (Kleinnijenhuis et al., 2014).

The SARS-CoV-2 infection renders hyper-inflammation mediated pulmonary dysfunction due to pro-inflammatory cytokine (IL-1β, IL-4, IL-6, IL-8, MCSF, CXCL-10, and TNF-α) burst in patients (Guo et al., 2020; Li et al., 2020). Surprisingly, these elevated cytokine levels have been reported along with lymphopenia in COVID-19 patients, suggestive of a major contribution by uncontrolled innate responses. The anti-inflammatory cytokine IL-10 could be of great help in combating COVID-19 because of its anti-inflammatory nature and its capability to obstruct inflammatory cytokine production, including IFN-γ and IL-2. BCG is shown to induce T and B independent monocytes/macrophages and NK cell-mediated nonspecific trained immunity response to a secondary infection by the innate immune system to either the same or different microorganisms (Covian et al., 2019; Netea et al., 2020) in SARS-CoV-2 infection in BCG immunized individuals. Moreover, it would be imperative to compare the cytokine profiles of patients from BCG immunized and nonimmunized individuals.

Thus, BCG induces sustained changes in the immune system associated with a nonspecific response to infections that could be beneficial against COVID-19. However, the beneficial role of BCG against COVID-19 remains debatable because of the variation in testing rate, population density, median age, TB incidence, urban population, public policy, and community spread check measures in different countries (Kirov, 2020). BCG as an adjuvant is proved very efficient in producing nonspecific immune responses; in the case of COVID-19, we should perform studies to evaluate the efficacy of various adjuvants, including BCG to elicit immunoprophylactic responses by candidate vaccines. The suitable adjuvant would not only instigate immune responses, but will also facilitate memory response that could be a boon for a vaccine against this dreadful pathogen.

## 6. Discussion and Future Prospects

The most daunting threat in a century for mankind is the COVID-19 pandemic caused by SARS-CoV-2. Less than a year after COVID-19 was declared as a pandemic, several vaccine candidates were authorized for emergency use in various countries. Despite the availability of vaccinations, recent pandemic waves have created concerns about i) the effectiveness of existing vaccines against new virus variants; ii) achieving global herd immunity against COVID-19; iii) the safety of the vaccine in autoimmune disorder individuals; iv) vaccine safety for pregnant women, toddlers, and young adults; v) the possibility of cytokine storm in vaccinated people; vi) efficacy of vaccines in comorbid conditions; vii) appropriate timing of booster doses for best immune response; viii) B cell memory responses; ix) T cell immunity and memory response; x) effectiveness of vaccines in reducing viral transmission; xi) vaccine distribution to the underprivileged; xii) the effect of vaccination on infected people; xiii) vaccine hesitancy; and xiv) vaccine swapping in the absence of a second dose or improving immune response, continuing exploration of significant scientific and policy challenges (Garcia-Beltran et al., 2021; Singh et al., 2021).

Multiple waves of infection place a huge burden on the state, doctors, and scientists in establishing a successful fight against SARS-CoV-2. Some of countries have achieved a sufficient number of vaccinations to curb the pandemic. In contrast, some countries confirm lowered vaccinations either due to unavailability of vaccines as per requirement or because of vaccine hesitancy. Because of the severe impact of the pandemic in terms of infection intensity and mortality, the vaccination drives in between this pandemic have reduced vaccine hesitancy among the population, and this has contributed to achieving herd immunity. Herd immunity generally requires a protective immune response in 60%–70% of the population attained either through infection with the virus or by vaccination. Global scientific coordination and partnership is the key to successful vaccination against COVID-19. It is particularly effective because nationalistic approaches to immunization will continue to raise issues such as vaccine development and production, pricing, allocation, and deployment, and only a global resource collaboration can fulfill these goals (Voysey et al., 2021).

The vaccines used are safe, but there are reports of safety concerns with some of the vaccines, which has caused havoc and has been a source of vaccine hesitancy. Scientific evidence on vaccine safety should be established not just in the general population, but also for pregnant woman, toddlers, and teens; people with comorbid diseases such as diabetes and hypertension; and those who have been infected prior to vaccination. Because most of the vaccines are based on neutralizing antibodies against the spike protein of SARS-CoV-2, details are available for neutralizing antibodies; however, T cell immunity remains untouched for most of the vaccine candidates. Furthermore, the B and T cell memory response has not been established for most of the approved vaccines. B and T cell memory responses and T cell–mediated immunity should be deciphered for all the used vaccines with immediate attention.

Globally, the spread of different variants has worsened the situation, and we need a vaccine with prophylactic efficacy to combat COVID-19. The multivariant vaccines might be more efficient in eliciting better and stronger immune responses for better protection against virus variants. We can use various platforms to achieve multivariant vaccines. Nonetheless, the direct virus could be employed to develop live-attenuated vaccines; however, there is always the possibility of reversal. One of the major concerns during SARS-CoV-2 infection is cytokine storm, which leads to various physiological damages, including pneumonia; pulmonary embolism; and blood clotting in various organs, including brain, gastrointestinal tract, kidney, and liver; also it results in ARDS. This is suggestive that unregulated immune response due to viral immune evasion strategies renders these damages. The cytokine storm could be controlled by inducing Treg cells and IL-10 cytokine strategically. Nevertheless, BCG has been proved to elicit nonspecific controlled immune response; it would be an inclusive strategy to develop formulations of rBCG by incorporating multiple antigens of SARS-CoV-2. The live-attenuated nature of rBCG could be helpful in generating long-term protection.

Interestingly, a number of studies are currently being conducted in many parts of the world on the use of two separate vaccines for better protection against SARS-CoV-2 and its variant form. According to a German study, immunizing with AstraZeneca’s ChAdOx1-nCov-19 vaccine as a first shot and BioNTech/BNT162b2 Pfizer’s as a second shot 10 weeks apart boosts the immune response. BNT162b2 induced significantly higher frequencies of spike-specific CD4 and CD8 T cells and, in particular, provided high titers of neutralizing antibodies against the B.1.1.7, B.1.351, and P.1 lineage of the virus (Barros-Martins et al., 2021; Callaway, 2021). There has been speculation that using covaxin as a first shot and AstraZeneca (covishield) as a booster shot helps to protect people better. However, further research is required on the interchangeable vaccine approach.

Considering a worse hit by multiple waves, it is suggestive that individuals should take the vaccines whichever they are available at their end to curb the disease severity even if it does not provide the best protection. Importantly, prophylactic vaccines against SARS-CoV-2 have great potential to prevent future pandemics. Nonetheless, we must continue to improve the present vaccine by increasing neutralizing antibodies, and a focus on multivariant vaccines that include T cell immunity would be beneficial in combating numerous variations of this dreadful pathogen.

## Author Contributions

MA conceived and designed the review drafting. WH, ZH, RA, KG, AG, NK, IA, and FA contributed in writing, preparation of figure and tables, and updating the review. All authors contributed to the article and approved the submitted version.

## Conflict of Interest

The authors declare that the research was conducted in the absence of any commercial or financial relationships that could be construed as a potential conflict of interest.

## Publisher’s Note

All claims expressed in this article are solely those of the authors and do not necessarily represent those of their affiliated organizations, or those of the publisher, the editors and the reviewers. Any product that may be evaluated in this article, or claim that may be made by its manufacturer, is not guaranteed or endorsed by the publisher.
